# Knowledge, attitude and behaviour towards the use of insecticide treated mosquito nets among pregnant women and children in rural Southwestern Uganda

**DOI:** 10.1186/s12889-017-4824-4

**Published:** 2017-10-10

**Authors:** Ivan M. Taremwa, Scholastic Ashaba, Harriet O. Adrama, Carlrona Ayebazibwe, Daniel Omoding, Imelda Kemeza, Jane Yatuha, Thadeus Turuho, Noni E. MacDonald, Robert Hilliard

**Affiliations:** 1 Institute of Allied Health Sciences, International Health Sciences University, P.O Box7782, Kampala, Uganda; 2 Department of Psychiatry, Mbarara University of Science and Technology, P.O Box 1410, Mbarara, Uganda; 3 Department of Information Technology, Uganda Christian University Mukono, P.O BOX 4, Mukono, Uganda; 4 Infectious Disease Research Collaboration, Tororo, P.O Box 7475, Kampala, Uganda; 5 Infectious Disease Research Collaboration, Mbarara, Kampala Uganda; 6 Dalhousie University, MicroResearch International and IWK Health centre, Halifax, Canada; 7 Hospital for Sick Children, University of Toronto, Ontario, Toronto, Canada; 8 Department of Educational Foundations and Psychology, Mbarara University of Science and Technology, P.O Box 1410, Mbarara, Uganda; 9 Department of Biology, Mbarara University of Science and Technology, P.O Box 1410, Mbarara, Uganda; 10 Department of Medical Laboratory Science, Mbarara University of Science and Technology, P.O Box 1410, Mbarara, Uganda

**Keywords:** Malaria prevention, Knowledge, Attitude, Practices, ITNs use, Children under five, Pregnant women, Isingiro district, Uganda

## Abstract

**Background:**

The burden of malaria in Uganda remains unacceptably high, especially among children and pregnant women. To prevent malaria related complications, household possession and use of Insecticide Treated mosquito Nets (ITNs) has become a common practice in the country. Despite the availability of ITNs, malaria remains a foremost public health concern in Uganda. We sought to explore knowledge, attitude, and behaviour towards the use of ITNs as a nightly malaria prevention strategy among pregnant women and children under five years of age in Isingiro district, Southwestern Uganda.

**Materials and Methods:**

This was a community based, descriptive cross-sectional study, in which households with children under 5 years, and/or pregnant women were enrolled. We used a structured questionnaire to collect data on participants’ understanding of the causes, signs and symptoms of malaria; use of ITNs to prevent malaria; attitudes and behaviours towards the use of ITNs. We also conducted key informant interviews (KIIs) to get in-depth understanding of responses from the participants. We analysed quantitative data using STATA version 12.Qualitative findings from the KIIs were transcribed and translated, and manually analysed using thematic content analysis.

**Results:**

Of the 369 households enrolled, 98.4% (*N* = 363) households had children under five. Most participants (41.2%, *N* = 152) were in the 21–30 age category (mean age; 32.2 years). 98.1% (*N* = 362) of the respondents considered ITNs a key malaria prevention strategy. The ITN possession rate was 84.0% (*N* = 310), of these, 66.1% (*N* = 205) consistently used them. 39% of the respondents did not have a positive attitude towards ITNs.

**Conclusions:**

Although 84.0% of the respondents possessed ITNs, many were not consistently using them. To this, there is need to engage all stakeholders (including cultural leaders, community health workers, religious leaders and the government) in the malaria prevention campaigns using ITNs through: a) government’s concerted effort to ensure universal access of right fit ITNs, b) end-user directed health education to emphasize positive attributes of ITN use, c) telling the ITN success stories to improve on the usage.

## Background

Malaria remains a major infectious disease in most developing countries. Uganda has the third largest malaria burden in Africa [1], and malaria accounts for high morbidity and mortality among pregnant women and children under five [2]. Malaria during pregnancy is linked to gestational anaemia that is associated with low birth weight, abortion, and miscarriage [3, 4]. To the country’s health and economy, malaria negatively affects productivity, adds to costs of care [5], and impacts negatively on the household income.This has compelled the government of Uganda and implementing partners like the Global Fund and the Roll Back Malaria initiative to try to maximize the use of Insecticide Treated mosquito Nets (ITNs) to mitigate the effects of malaria among vulnerable populations. In addition, the World Health Organization-Global Malaria Program encouraged global efforts to fortify the use of ITNs for malaria prevention [2]. The use of effective ITNs are considered key to the elimination of the mentioned advance sequalae among the vulnerable populations.

As a result of these concerted efforts Ugandan household ITN possession rate rose from 47% in 2009, to 60% in 2011 and 90% in 2014 [6]. It is anticipated that ITN possession will rise ensuing governments programs to implement regular replacements. Although ITN use has proven to be effective in prevention of malaria [7, 8], end user acceptability remains a prime challenge [9]. In ITN utilisation surveys conducted in Tanzania and Nigeria, only a few respondents had ample knowledge about malaria and few of the respondents were using ITNs [10, 11]. On the other hand, attitude towards ITN utilisation is influenced by socio-cultural expectations and cultural beliefs about symptoms of malaria like fever, backache, nausea, loss of appetite and vomiting being regarded as signs of pregnancy, thus precluding married women from using those ITNs [12]. The knowledge barriers ascribed to use of ITNs for malaria prevention coupled with the perceived misconceptions and negative attitude towards use of ITNs as a prevention measure has led to misuse of ITNs [13]. Therefore, given the high rate of household possession of ITNs in Uganda, yet the continuing high rates for malaria, the aim of this study was to explore the knowledge, attitude, and behaviour towards the use of ITNs as a malaria prevention strategy among pregnant women and children under-five in Isingiro district, Southwestern Uganda.

## Methods

### Study site

We conducted the study in Isingiro district, located in rural south western Uganda. Isingiro district is about 297 Kilometres from the capital city- Kampala, and 47 Km from Mbarara town. It has 14 sub counties and 3 town councils. The district has a population of 492,116; of whom 254,567 are females [14]. Malaria is a frequent and serious problem; 96 confirmed cases were reported in the 4th quarter of 2014 alone [15].

### Study design

A community based, descriptive cross-sectional study was carried out during the months of July to November, 2015.

### Study respondents, sample size estimation and recruitment

We planned to enrol respondents from Isingiro district who had children under5 years, and/or a pregnant woman, whose family head was 18 years or older at the time of the survey based upon a sample size calculation formula [16]. Assuming a 95% confidence interval, ITN coverage estimated to be 60%, [17] and the maximum allowable error at 5%; 369 households were considered. Our study unit consisted of two villages in each sub-county. In a village, we purposefully chose ten households from village major points including a church, school, health unit, trading unit and a bridge. For each household, a respondent who was a household head, a spouse, or senior matriarch was selected to be interviewed using the structured questions. In the field, we purposely selected 15 people for key informant interviews (KII) including local council leaders, district health inspector, religious leaders, health workers and members of village health teams (VHTs). VHTs serve as the community’s primary health contacts, the equivalent of the lowest-level health center and it’s the initial point of care. These were selected because their community responsibilities allowed them to interact with many people and thus have broader information regarding the use of ITNs.

### Data collection instruments and method

We collected data between July and November 2015. We used a structured questionnaire and KII guide that were developed from earlier studies based on malaria and ITN usage [7, 18–20]. These were developed in English, translated to Runyakore, back translated to English and checked for content. The integrity of translations was ensured by the corresponding author (IMT) and three of the research assistants who were well versed with both languages used in the study.The questionnaire comprised 25 mostly closed questions in four sections;

1) socio-demographic data: gender, age, level of education, household source of income, number of occupants in a household, and the number of children in the household who were under five years; 2) basic knowledge about malaria particularly the causative agent(s) of malaria, common signs and symptoms of malaria infection, ways to prevent malaria and use of ITNs to prevent malaria; 3) attitudes about the use of ITNs: including respondents’ thoughts on use of ITNs by pregnant women and children under five to prevent malaria and 4) behaviour towards the use of ITNs: probed for frequency of ITN use, and if ITNs were used by every member in the household. In addition, there were questions that probed respondents’ likely reasons for non-possession and non-use of ITNs, and attributable gains among those who consistently used ITNs.

The questionnaire and key informant interview guide was pilot tested on thirteen households and two informants respectively who were not part of the main study, and changes were made accordingly. The questionnaires were administered by trained research assistants who were fluent in both local language (Runyankore) and English. Questionnaires were checked for completeness and corrected when typographic or comprehension errors were found.

### Data analysis

Data were organized using EpiData3.1 and transferred to STATA 12.0 for analysis. Descriptive statistics (frequencies and percentages) were used to tabulate and describe data. Primary analyses identified correlates of self-reported socio-demographic data which included gender of household head, participant’s age, level of education, number of children under-five, total number of occupants in household, the basic knowledge about malaria and ITN use. Participants’ basic knowledge on malaria was based on the seven questions that embodied malaria causation, signs and symptoms of malaria infection, ways to prevent and control malaria, source of information on malaria prevention, household possession of an ITN and its consistent use.

The audio recordings from the KIIs were translated and transcribed from Runyankore into English. Two of the authors assisted by one experienced qualitative researcher used thematic content analysis to organize themes from qualitative data. Qualitative data was analysed manually.

### Ethics approval and consent to participate

We received ethical approval from Mbarara University of Science and Technology Research Ethics Committee (REC). All respondents provided written informed consent after receiving detailed description of the study. Eligible participants were consented in privacy and no incentives were given. Anonymity of the respondents was ensured at all stages of data analysis.

## Results

### Socio-demographic characteristics of study participants

We enrolled 369 respondents and of the 382 households approached, 96.6% agreed to participate. The majority (98.6%) of the households had children under age 5 years. Details of the demographic data are presented in Table 1. The majority of the respondents were female (64.0% *N* = 236) and the mean age of the respondents was 32.2 years (range 18–68 years); over half (57.5%) had had attained primary level of education.

**Table 1:** Socio-demographic characteristics of study participants (*N* = 369)

Variable	Frequency, N	Percentage, %
Category of the respondents		
Females	236	64.0
Males	126	34.1
Matriarchs	7	1.9
Sex of household head		
Females	134	36.1
Males	235	63.9
Participants’ age category (Years)		
18–20	27	7.3
21–30	152	41.2
31–40	131	35.5
41–50	36	9.8
Greater than 50	23	6.2
Level of education		
No formal education	58	15.7
Primary	212	57.5
Post Primary	99	26.8
Source of income		
Farming	223	60.4
Small scale business	53	14.4
Government or NGO employees	93	25.2
Number of people in a house hold		
1–3	102	27.6
4–6	194	52.6
Greater than 6	73	19.8
Number of children under five in a household (N = 363)		
1- 2	316	87.1
2- 4	47	12.9

### Knowledge about malaria

Majority of the participants (83.3%, *N* = 305) were aware that malaria is transmitted by mosquitoes. Almost all (98.6%, *N* = 364) could recognize the common signs and symptoms of malaria infection. All participants mentioned one sign/symptom of malaria. Almost all participants knew that ITN use can help prevent malaria (98.1% *N* = 362). (See Table 2).

**Table 2:** Knowledge about malaria, causes, symptoms and prevention

Variable	Frequency (*n*)	Percent (%)
Causes of malaria (*n* = 366)		
Mosquitoes	305	83.6
Bedbugs	43	11.7
Cats	10	2.7
Rats	8	2.2
Signs and symptoms of malaria infection (*n* = 364)		
Fever	222	61.0
Headache	23	6.3
Chills	7	1.9
Energy loss	36	10.0
Sweating	75	20.6
Vomiting	1	0.3
Ways to prevent and control malaria (*n* = 365)		
Sleeping in bed nets	362	98.6
Clearing bushes around homesteads	1	0.3
Using insecticides to spray around and in homes	1	0.3
Wearing long sleeved clothes	1	0.3

### Possession and utilization of ITNs

The ITN possession rate was 84.0% (*N* = 310). On the other hand, 16.0% (*N* = 59) neither possessed, nor used an ITN. Of the 84.0% who possessed ITNs, only 66.1% (*N* = 205) consistently used them for children under five and pregnant women and those in possession but didn’t use the ITNs were 33.9%. Those who did not use the nets and the households that didn’t possess (16.0%), gave varied reasons as summarized in Tables 3 and 4 respectively and these reasons did not vary by village.

**Table 3:** Reasons for ITN non-possession among respondents (*N* = 59)

Variable	Frequency	Percent (%)
Not received	21	35.6
Uncomfortable to use	14	23.7
Destroyed	4	6.8
Lack of information	7	11.9
See no benefits	13	22.0

**Table 4:** Reasons for ITN possession, but non-use among respondents (*N* = 27)

Variable	Frequency	Percent (%)
Side effects	7	25.9
Poor quality ITNs	14	51.9
Worn-out ITNs	2	7.4
^a^False beliefs	4	14.8

^a^False beliefs among respondents included: the effect of chemical within the ITN causes cancer, use of mosquito nets complicates breathing and the fact that malaria is linked to pregnancy therefore use of ITNs may not impact on malaria prevention among pregnancy

### Reasons for ITN possession, but non-use among respondents

Despite possession, some of the respondents did not put the mosquito nets to use. Reasons advanced for this varied between respondents (Table 4). For example, almost 41% of respondents who were not using ITNs (*N* = 11) were concerned about side effects of the ITNs or had beliefs that chemicals used could cause cancer.

### Qualitative data

#### Knowledge about malaria

Respondents were aware that malaria is caused by mosquito bites as exemplified in the following quote:

*Mosquitoes feed on human blood and malaria is transmitted by mosquito bites during the process of feeding’. (36 year old, pregnant woman)*

In addition, respondents could recognize the signs and symptoms of malaria, as they listed some or all as signs and symptoms of malaria as given in the narrative:

*Infestation with malaria is associated with shivering, joint pains, body weakness, body pain, dizziness, vomiting, and loss of appetite, sour mouth and sores in the mouth.*

The healthcare respondents, in addition to the signs and symptoms already mentioned;

*Failure to suckle for children, sunken eyes as a sign of dehydration, head-and stomach aches and generalized body pains.*

Respondents indicated that pregnant women, children under five, people living with HIV and Acquired immune deficiency syndrome (AIDS) as well as the elderly were the most at risk population as quoted:

*‘Unlike the previous ITN distribution programs that were targeting pregnant women and children (during ante-and post-natal care visits) and people living with HIV and AIDS (during their clinic visits), this time the ITN exercise has covered every household member irrespective of such factors’. (Local council chairperson of V_SubCounty)*

### Attitude towards use of ITNs

Respondents gave varied responses concerning use of ITN use for malaria prevention. Of those agreeing that use ITN use helps to prevent malaria (333 of total respondents), 225 strongly agreed. This respondent strongly urged the vast benefit of ITNs as narrated:

*‘I (respondent) have witnessed moments where my friends (perhaps more would testify) that lives of pregnant women and children have been saved from malaria which had earlier posed a threat to our community’. (Man 38 year old)*

Another respondent adds:

*‘In this village, majority of the homes that use ITNs rarely fall sick of malaria. I have spent four years using a mosquito net, and during this time, whenever I fall sick and go to the hospital, am found not to have malaria, and treated for other diseases’. (Woman 24 years old)*

*‘For me (respondent) every two months I used to have a child hospitalized for malaria, but since I started using the mosquito nets, malaria has reduced in my family’. (Man 29 father of two)*

### Behaviour towards the use of ITNs

Respondents gave varied explanations on how they used the ITNs and what they did to ensure effective use. The majority of the respondents (77.1% *N* = 239) were well versed with the basics of appropriate ITNs usage. These are quotes from some of the participants about usage at night; however, note the in appropriate washing in the second quote:

‘*I began by leaving it (ITN) hanged outside for three nights (may be to reduce on the smell) as we were told by VHTs, then, using the sisal threads, I suspended the corners of the net above the bed, and fit it to cover the four corners of the bed. At night, I draw the net, enter the bed and cover it fully to deter away mosquitoes’. (Pregnant woman 27 years old)*

*I washed the nets and dried it (ITN) under sunlight. I then fixed the four corners of the net into the four bed poles and pulled the net to cover the mattress. Every morning I leave the bed covered to prevent mosquitoes from entering.*

### Benefits of the use of the ITNs

Among the households in possession and reportedly used ITNs consistently, there were varied benefits reported according to different respondents. Some of the respondents indicated improved health status and socio-economic wellbeing as indicated by respondents below:

*‘Malaria has reduced in my home; most neighbours have taken time without suffering from it [malaria], and at some point we think that malaria is out of this area’. (Man 33 years old, father of three)*

As malaria affects all age groups, a respondent highlighted how the use of ITNs has markedly improved on children’s school attendance:

*My children no longer fall sick of malaria and this has enabled them to attend school regularly*. *(Man39 years old)*

Whereas malaria-like symptoms may manifest in other illnesses, one of the KIs stressed the positive attribute of ITN usage in his home as:

*I have spent about 11 years using ITN, and all this time, whenever we (household members) fall sick and go to the hospital to test for malaria, it’s found negative. We are then treated for other malaria-like illnesses. (Man46 years old)*

### Factors associated with non-use of ITNs

The factors associated with non-use of ITNs did not vary by village. Although most respondents possessed ITNs, their utilization was hindered by factors related to ITN quality, size and color. Some respondents stressed displeasure as highlighted in the quote:

*They (ITNs) create warmth causing over sweating’. (23 year old, pregnant woman)*

Some respondents reported ITN texture as a hindrance to their use some users as quoted:

*It’s too rough; the roughness is like a cow’s hide, and when it (ITN) touches on a baby’s skin, it causes a bruise. (32 year old, mother/nurse).*

Additionally, the sizes of the ITNs were unsatisfactory, and could have therefore hindered their effective use as indicated by one of the respondents in the following quote:

*The nets are too short to cover the bed, and in most cases I find my baby when he isn’t covered. (Woman 28 year old)*.

The concentration of insecticide in the ITNs was also an issue as raised by a respondent:

*Their (ITNs) chemical has an irritating smell, and most users believe that the effect of this[chemical] to mosquitoes could as well cause cancer in humans. (41 year old, father of five).*

As color is a choice of demand, some respondent expressed concern as stated in this narrative:

*‘The colour of white that was distributed shows a lot of dirtiness for us whose houses were constructed out of mud, without a ceiling and I still use local paraffin candles as a source of light. I would have preferred a color which may hide dirt and smoke’. (30 year old, pregnant woman)*

Amongst those who had ITNs but did not use them for sleeping the most common diversions reported during the field survey were:

*‘In our communities, a number of people have used them for rearing chicken, since they (people) did not find them fit for human use’. (47 year old, Chairman Local Council 1).*

*‘Among fishermen they use them (ITNs) to catch and dry fish’. (28 year old, VHT)*

## Discussion

Our study findings indicate that although most of the respondents in the rural areas have ITNs (84%) and are knowledgeable about the causes, atleast one of the signs and symptoms of malaria and the value of ITN use as a preventive measure (over 90%), only 66.1% were using them regularly. The ITN self-reported possession of 84.0% is noble as it’s slightly higher than the WHO reference of at least 80% [2], and this is ascribed to the recent mass rollout by the government [17], and also vertical equity where the most at risk populations (pregnant women and children under five) receive the best protection against malaria. Knowledge, that ITNs can decrease malaria was not enough to convince all who had ITNs to use them. However, 16% either did not possess an ITN and 33.9% were sceptical about their use due to perceived concerns such as possible negative effects of the chemicals in the ITNs, risk of suffocation with ITNs or dangers such as cancer for pregnant women and children. Previous research had similar findings with people not using the freely distributed ITNs due to factors linked to adverse risks of the insecticide [17–19].Among those who did not use them, some (*N* = 4) also undermined the benefits by washing the ITNs because of concerns about the smell and the effects of chemicals used on children and pregnant women. The respondents were knowledgeable on how to correctly use the ITNs despite negative sentiments towards their use. Despite the majority strongly commending use of ITNs due to the immense benefits in their communities they had concerns about the poor sizes, roughness and colour choice of the ITNs distributed by the government, which is thought to have undermined ITN use.

Our study findings are in agreement with previous studies that most people in Uganda have ample knowledge about the causes of malaria, signs and symptoms and the available preventive strategies such as ITNs [21]. Moreover previous studies have suggested that having knowledge about the causes of malaria, signs and symptoms of malaria would enable people seek care and intervention appropriately [11, 22–24]. Other studies have reported non-use due to similar concerns noted in our findings (7, 18–19).The misuse of ITNs to inappropriate use like fishing and poultry farming is consistent with findings of previous research [25].

Our results should be interpreted in light of the following limitations. The use of ITNs by children under-five and pregnant women was analyzed only for the night prior the survey and may therefore, not reflect the long-term pattern of ITN usage. Although these ITNs had been distributed in less than a year to the time of the study, it’s likely that the insecticide may have lost potency; as we didn’t ask questions to ascertain accurate assessment of ITN status, this may over rate the prevalence of ‘effective ITNs’ and their use. Whereas some of our respondents reported ITN non-use due to poor quality, color and roughness; the study did not assess for such aspects therefore we may neither ascertain these as hindrances to ITN use nor facts to ignore. The study targeted household head as a proxy to the knowledge, attitudes and behaviours of the entire household. Thus, results may not accurately represent their perspectives as a whole. Our research tools captured self-reported information and relied mainly on respondents giving the right information, it’s likely that self-reported use of ITNs may have introduced bias.

## Conclusions

Our study findings indicate that the majority of the people are aware of the causes of malaria, different signs and symptoms of malaria and preventive strategies including consistent and appropriate use of ITNs for sleeping. However the high rate of ITN possession and good knowledge did not translate into overwhelming usage and this impact negatively on the prevention of malaria among vulnerable populations. Three major factors appeared to influence non-use a) false beliefs and concerns about adverse risk of chemical in the ITNs, b) ITNs of poor size and c) lack of access to ITN. Thus a multi-pronged approach is needed to enhance use. Firstly, the government both centrally and locally needs to ensure universal access and this should be for ITNs that are big enough to fit the beds. Replacement of ITNs on a regular basis needs to be part of the program. Secondly directed health education to emphasize the positive attributes of ITNs towards malaria prevention needs to be carried out at the local village level likely through the village health teams; including education on the added value of the insecticide for protection i.e. not prewashing the nets before use. Furthermore telling the stories of benefit noted in the quotes above may make this behaviour more socially normative and promote resiliency of use in more village homes. As indicated in previous studies [26–28] addressing disparities that impact negatively on the use of ITNs will improve on further usage of ITNs and prevent or eliminate malaria from the communities. To best address the gaps in use, all stakeholders should be involved and engaged in the malaria prevention campaigns including cultural leaders, community health workers, religious leaders and the government.

## Abbreviations

ITN: Insecticide Treated Mosquito Net
KII: Key Informant Interview
VHT: Village Health Team.
